# Tukey’s Warning and Pascal’s Wager

**DOI:** 10.1007/s42113-026-00322-7

**Published:** 2026-09-15

**Authors:** Kenneth J. Malmberg, Amy H. Criss

**Affiliations:** 1 Department of Psychology, University of South Florida, Tampa, FL 33620 USA; 2 Department of Psychology, Syracuse University, Syracuse, NY 13244 USA

## Abstract

We comment on an article by Shiffrin, Stigler, and Keil (2026) relating illusory beliefs of understanding to hubris, discussing the dangers associated with accepting a model as a lawful fact and the benefits of questioning one’s models.

*Hubris is the greatest danger that accompanies formal data analysis* - J.W. Tukey.

Shiffrin, Stigler, and Keil suggest people are susceptible to illusions of understanding. They describe several hypotheses for illusions of understanding in science and how they affect scientific progress and translation to societal impacts. However, many disagree with their controversial thesis that ‘All models are wrong; some models are useful.’ This essay will not produce a consensus by itself, if for no other reason than models are inherently flawed approximations for some, but a model is something akin to a law for others. In psychology, for instance, we have Fechner-Weber’s Law, but this law is an empirical regularity, much like the Law of Gravity. These highly accurate descriptions of nature are useful but do not explain what gives rise to the natural phenomena they predict as we argue below. Recent essays on the cumulative nature of scientific progress and the limitations of explanation by Navarro (2021) and Fried (2020) draw a similar conclusion.

For some, the lawful regularity of a phenomenon implies perfect understanding, while others are inspired to conduct further research to address what remains unknown. When Niels Bohr was asked a question concerning the explanatory power quantum mechanics, he often stated, “There is no quantum world. There is only an abstract quantum physical description. It is wrong to think that the task of physics is to find out how nature is. Physics concerns what we can say about nature,” according to Bohr’s assistant (Peterson, 1963). Accordingly, what we can say about nature is probably different from nature in reality, and advances in our understanding of quantum mechanics, or any field of study, are still being made precisely because there are no perfect models.

Scientific inquiry never ends, and some find this unsettling, perhaps due to their hubris. A tonic to treat this particular distress is simple if we take the view that models and laws are not equivalent. On this, the goal of modeling is to provide a concrete expression of one’s point of view along with relevant observations, those that are supportive and those that are not if they are known, and the most we can do is conclude the point of view meets some arbitrary set of standards, which may change over time as new observations are collected. Rigorously testing models can give us confidence that they might pass additional tests, but confirmation does not imply that a model is correct.

Falsification of concrete models is often easy. To wit, several models are abandoned by the scientist on the way to a model that they find satisfying. Putting a model through a series of tests, may contribute to an illusion that the model and reality are the same. However, the fact one stops refining their ideas provides no indication that the model is correct. Indeed, the stopping rule is often unspecified. Even when an arbitrary ‘significance’ test is used, the scientist may have found a better model, if so motivated or if the data were less noisy, if the past is an indication of the future.

There is no harm in acknowledging the limitations of one’s knowledge. A humbler approach to science does not limit the utility of the models scientists create. There are many examples of imperfect models greatly improving the human condition.

Those living on the Gulf of Mexico begin visiting spaghettimodels.com beginning in the summer every year. The term *spaghetti models* is a layperson’s euphemistic way of referring to a collection of hurricane tracks different models forecast; when plotted together they look like a plate of spaghetti. Thus, the models differ, and none are correct. When jointly considered, however, the National Hurricane Center generates a *cone of uncertainty* estimating where there is a 75% chance that the center of the eye of a hurricane will make landfall. As the NHC forecast extends into the future, uncertainty in the future position of the hurricane increases. Last year, TS Milton formed off the east coast of Mexico 72 hours in advance of making projected landfall, and the initial center of the cone of uncertainty differed from the actual point of landfall by less than 10 miles. Hence, the flawed tracking models have tremendous positive impact by providing guidance that reduces but does not eliminate uncertainty. The NHC explicitly warns that each forecast is uncertain, as are all forecasts. Citizens who assume the forecast is correct have a false sense of security. Hubris can lead to deadly practical outcomes.

We can think of other examples in our own domain of research. Since the earliest days of psychophysical research we have observed that one can accurately determine whether two physical stimuli are identical or not. The ability seems quite systematic, and Fechner’s and Weber’s Law relate various phenomena. But we do not understand how one decides if two simple stimuli match (cf. Goldstone, 1994). Likewise, many memory models assume that feature matching is the basis for the performance of memory tasks, and likewise we have no idea how this takes place. We have a computer do this using an arithmetic logic unit that subtracts one binary representation from another in order to determine the difference. Yet, the computer will not naturally produce data consistent with psychophysical laws. On this, there is no reason to be certain models that rely on matching are correct, and believing that a model is reality discourages one from investigating what we do not understand and questioning what we believe we know. Hubris slows progress.

The proposal to focus on the utility of models rather than their correctness is somewhat reminiscent of Blaise Pascal’s wager, who was troubled by the ability of his probability theory to predict the outcome of future events, which until that time were attributed to God’s will. How could a religious person reconcile his belief in his theory and his belief in God’s will? Pascal said (in short), its better to believe in God should it not exist, than to not believe in God should it exist (Pascal, 1932). We can extend this idea: It is better to question a model if it is correct, than it is to trust a model if it is wrong. And all models are wrong.

## Data Availability

No datasets were generated or analysed during the current study.

## References

[CR1] Fried, E. I. (2020). Lack of theory building and testing impedes progress in the factor and network literature. *Psychological Inquiry,**31*(4), 271–288. 10.1080/1047840X.2020.1853461

[CR2] Goldstone, R. L. (1994). Influences of categorization on perceptual discrimination. *Journal of Experimental Psychology: General,**123*(2), 178–200. 10.1037/0096-3445.123.2.178 8014612

[CR3] Navarro, D. J. (2021). If mathematical psychology did not exist we might need to invent it: A comment on theory building in psychology. *Perspectives on Psychological Science,**16*(4), 707–716. 10.1177/1745691620974769 33593197

[CR4] Pascal, B. (1932). Pensees. Translated by Warrington, John. available in .pdf from UCLA.

[CR5] Peterson, A. (1963). The philosophy of Neils Bohr. *Bulletin of the Atomic Scientists,**19*, 8–14.

[CR6] Tukey, J. W. (1986). Sunset salvo. *The American Statistician,**40*(1), 72–76.

